# Newly Qualified Nurses’ Perception of Their Competency Achievement on Leaving University: A Qualitative Study

**DOI:** 10.3390/ijerph16214284

**Published:** 2019-11-04

**Authors:** Olga María López-Entrambasaguas, Rocío Martínez-Yebenes, María José Calero-García, José Granero-Molina, José Manuel Martínez-Linares

**Affiliations:** 1 Nursing Department, Faculty of Health Sciences, University of Jaén, Campus Las Lagunillas s/n, 23071 Jaén, Spain; rociomarye92@gmail.com (R.M.-Y.); mjcalero@ujaen.es (M.J.C.-G.); jmlinare@ujaen.es (J.M.M.-L.); 2 Nursing, Physiotherapy and Medicine Department, Faculty of Education Sciences, Nursing and Physiotherapy, University of Almería, Carretera de Sacramento s/n, 04120 Almería, Spain; jgranero@ual.es; 3 Faculty of Health Sciences, Universidad Autónoma de Chile, Santiago 4780000, Chile

**Keywords:** qualitative research, students, nursing, nurses, competency-based education, education, nursing

## Abstract

*Background*: After implementing the Tuning Educational Structures in Europe Project, numerous efforts have been made to define, establish, and evaluate nursing competences. The European Federation of Nurses Association played a key role in enacting the nursing competences included in Directive 2013/55/EU. Nevertheless, assessing competences remains elusive, and there is little research into nurses’ perceptions of the competency training provided by their universities. The purpose of the study was to explore the perceptions and experiences of newly qualified nurses about the competences they acquired during their university education. *Methods*: A qualitative research study was developed in a Spanish university. Twelve semi-structured interviews with newly qualified nurses were conducted, and two focus groups made up of twelve students were carried out in order to triangulate the results. Participants were recruited through email contact. Interviews focused on clinical training, theoretical content, and the reality of healthcare. Data was analyzed thematically. *Results*: Two main themes emerged: (1) improving theoretical content and (2) rethinking practical lessons and clinical training. A lack of knowledge about mental health, pharmacology, or critical care has been found; in addition, it was highlighted, among others, the need to improve communication skills in difficult and conflictive situations. *Conclusions*: Considering the participants’ perception of deficiency in some aspects of most of the competences established by the European Federation of Nurses Association, further research has been suggested to include other stakeholders’ views.

## 1. Introduction

The convergence of European Higher Education has been defined as a globalization of knowledge [1] in Europe. The path to achieving a comparable, prestigious, and internationally recognized higher education in Europe [2] lies in aligning key aspects of the academic curricula of the 48 member states. To contribute to the development of these goals, the Tuning Educational Structures in Europe Project (TESE) [3] agreed to homogenize the activities that the former would be capable of performing. These were then divided into generic and subject-specific competences.

In 2005, six groups of specific competencies were established for an undergraduate degree in nursing [4]. Spain, for its part, consolidated the new nursing curriculum and competences in the Nursing Degree White Paper [5], which identified 40 specific competences [6] and grouped them into the same six categories established in the TESE [4]. Three years later, the Spanish government published regulations on the nursing profession [7], enshrining the competences that a nursing student must possess after completing his or her bachelor’s degree.

On the other hand, the most recent binding legislation relating to specific nursing competences can be found in the EU directive 2013/55/EU [8]. The European Federation of Nurses Association (EFN) gathered together the eight competencies specified in the directive and the six competency areas proposed in its Competency Framework [9], resulting in a set of guidelines to be used when creating nursing curricula in Europe. In turn, the TESE [4] states that quality should be guaranteed in higher education, taking into account the contribution of students and stakeholders while analyzing, developing, and maintaining educational programs—a statement that is intrinsically connected to competency acquisition.

The term “competence” (or “competency”) has created a certain degree of confusion in academic and professional spheres [10]. In any case, competence has been directly linked to employability [10,11] and alludes to the acquisition of skills in three spheres: knowledge, execution, and attitude [12]. Likewise, professionals must show certain attitudes and values [11] and use their knowledge in complex and diverse contexts, making use of the resources at their disposal [9].

Despite the importance given to the acquisition of competences, and the efforts made at an international level to reach a consensus, the method of assessing them remains an issue [13,14]. A variety of quantitative investigations have been carried out to approach this matter [14,15,16,17,18,19]. A mixed-method approach was developed in a Spanish university to evaluate the achievement of practicum competencies [20]. However, there is a lack of qualitative research into the experiences and perceptions of new graduates about whether the theoretical and practical training they received at university competently prepared them to face the reality of work.

An absence of qualitative studies in relation to this topic has been identified. Consequently, in order to fill this knowledge gap, taking into account the EFN Competency Framework and the recommendations to guarantee higher education quality [4], the purpose of the present paper is to explore and comprehend the perception of newly qualified nurses (NQN) about their competency training and acquisition after they have finished studying at university.

## 2. Materials and Methods

### 2.1. Study Design

A large, exploratory and descriptive qualitative study was designed. One of the aims of this project and its results are reported here. Qualitative research is appropriate in order to investigate unexplored areas [21] and enables the understanding of nurses’ perception of their competency achievement.

### 2.2. Participants

Purposive sampling was adopted and graduate nurses who had completed their bachelor’s degree in this university were recruited. Two inclusion criteria were established: (1) having between 3 and 12 months’ work experience as a clinical nurse in Spain, and (2) having graduated in the years 2017, 2016, or 2015.

Potential candidates were contacted by the main author via e-mail; the message included a brief description of the project and the inclusion criteria, as well as information on data collection, anonymity, and confidentiality. A consent form with full disclosure of the study was sent to those nurses who had emailed back to show their interest in participating. The dates and times for the interviews were agreed by email and the signature of the informed written consent was obtained before the data were collected. Besides, they were verbally informed that the Ethics Research Committee of the university had approved the study. The definitive sample was made up of 12 nurses once rich data were obtained and “characteristics within categories” were saturated [22].

Volunteering fourth-year students were selected and divided into 2 focus groups (FGs) in order to triangulate some of the results obtained from the individual interviews. The method of approach, information, and consent procedure was the same as for the NQN. A total of 12 fourth-year students participated, i.e., 2 FGs with 6 participants in each one.

### 2.3. Data Collection

Data collection was carried out in two phases. In Phase 1 data were collected between March and April 2018 through semi-structured, face-to-face interviews. They were held in a university meeting room. For three participants who were geographically dispersed, the interviews were conducted via Skype.

The interview guide was drafted after a review of the literature (Table 1). It was eventually configured around 3 pre-specific categories: clinical training, theoretical training, and the reality of healthcare (Table 2). The interviews lasted between 45 min and 1.5 h, and were audio recorded and later transcribed.

In Phase 2, FGs were carried out in September 2018, once data from individual interviews had been analyzed, in order to triangulate some results. FGs were performed by two students who were collaborating with the research group to which the researchers belong. The researchers did not perform the group interviews themselves in order to minimize bias, as they had been teaching the participants the previous year. The FGs lasted between an hour and an hour and a half, and were audio recorded and later transcribed. The questions asked were related to those areas in which the researchers had found possible shortcomings in the educational curriculum (Table 3). Fourth-year students were selected to be part of the study with the purpose of verifying if those supposed curriculum weaknesses were still occurring.

### 2.4. Data Analysis

A thematic analysis of the data was performed. Atlas.ti. 7 software was used to manage the data. Two of the researchers independently analyzed the transcriptions, following the 6 steps presented by Braun and Clarke [26], and met after Step 2 to ensure that the coding was the same. A second meeting took place with a third researcher to define and name themes. The complete process of ensuring trustworthiness is described in Table 4, as stated by Nowell et al. [27].

### 2.5. Ethical Considerations

The Ethics Research Committee of the university had approved this study (JUL.18/1.PRY). The first author informed the participants about the goal of the study, the voluntary character of their participation, and the anonymity and confidential treatment of the data. All participants provided written and verbal informed consent. Confidentiality and anonymity were assured throughout. In addition, participants were informed that they could withdraw from the study if they so wished.

## 3. Results

Two themes emerged from the qualitative analysis and were further supported by subthemes, which are described below with illustrative quotes from participants. Similarly, the results obtained are framed within the competency areas set out by the EFN Competency Framework [9] (Table 5).

### 3.1. Need to Improve Theoretical Content

In response to the question “Are you satisfied with what you learned on a theoretical level on the course?”, all of the responses could be summarized as “On the whole, yes”. Therefore, this theme relates to the lack of certain specific competences and areas of knowledge during their time at university.

#### Shortcomings of Academic Competences

We found that the limitations in theoretical knowledge were due to three factors: There was not a course that provided it; the theoretical content was insufficient or inadequate; or the theory taught had little practical applicability, according to participants’ comments.

• Deficiencies due to absence of course

One area of care in which some of our participants expressed a lack of knowledge is with respect to caring for a critical patient. This content is absent from the curriculum:

“My practicum was in an intensive care unit, and when I went in I couldn’t believe my eyes…I thought, ‘this is a completely different world’. It’s crazy, there are dozens of techniques that we’d never seen before, (…) I think a dedicated course on this subject would be a huge help because we haven’t got a clue about it.”.

(FG 1—Student 3)

• Deficiencies due to insufficient content

All of the participants agreed on feeling inadequately competent regarding mental health conditions. They expressed their dissatisfaction with the content of the course that they had taken about mental health:

“We didn’t learn what schizophrenia is, for example, or depression. If you talk to other people they’ll tell you the same—we didn’t learn anything…it was a pity”.

(I3)

The nurses interviewed and the students in the focus group also agreed about the teaching on pharmacology. They considered this to be a core course for this profession and that it needed to be taught for more than one semester.

*“Pharmacology should be annual. When you start your practicum or start working, you realize you’re missing things. You only have four months to study all of pharmacology, and obviously you do study it, but…you forget it*”.

(I5)

Participants reported a lack of knowledge on theoretical aspects that were very significant, such as wound care, including the treatment of pressure ulcers:

*“What have you learned at work that you didn’t learn during the degree program? All about wounds, ulcers, and other (similar) things. That’s where I came out of my degree very green*”.

(I10)

• Deficiencies due to course content with poor applicability

The participants expressed that some theoretical contents of the course could not be applied to the reality of healthcare. They consequently saw them as having little importance, for example those related to cultural competences:

“It would be better if we learned about cultural aspects that are more closely related to our career, perhaps on endogenous illnesses…It should be about how this or that pathology is treated in different places, and how those people are accustomed to a different kind of healthcare…”

(I6)

We also found some participants who made no negative comments on their theoretical training:

“I am satisfied with theory learned in class. Because I have met people who have studied in other Spanish universities or other countries and when you share experiences regarding classes or clinical practices… I think this university is quite good”.

(I5)

### 3.2. Rethinking Practical Classes and Clinical Training

From the discussions, the need to strengthen the teaching of activities and practical tasks of the profession in certain respects can clearly be inferred:

“You don’t learn what nursing is in class; you can learn the theory, but the most important place, where you learn the most, is in practice. In a few months we’re going to be entering the labor market and I’m not ready”.

(FG 2—Student 8)

#### 3.2.1. Need for More Practice

Most of the participants reported their frustration at having wanted to do more practice relating to communication with patients and families at emotionally difficult times. This nurse recalls his first days working in an oncology unit:

*“At first I felt I didn’t know what to say…I would go into the room, do whatever was necessary, but I went in as little as possible, to avoid being in there*”.

(I8)

The participants felt they knew about leadership fairly well but, as some of them stated, only on an “abstract” level:

“They told us that some people are born to be leaders, but that it can be taught; that’s what they said, but then nobody taught us how to be a leader”.

FG 1—Student 2

It was also found many reports of satisfaction with the learning of a variety of nursing techniques:

*“Did you practice all the nursing techniques?—Yes, I think so, even I channel subcutaneous routes, sutures, wound healing, pressure ulcers or catheterizations* …”

(I6)

#### 3.2.2. Insecurity upon Entering the Workforce

Some of the participants reported not having studied the skills necessary to work in an inter-professional team and having trouble dealing with certain difficult, conflictive situations:

“If a patient has wet or soiled themself and needs to be changed, I tell an auxiliary nurse and they say ‘I’m taking a rest’ or ‘I’m replenishing materials’. And I think: I don’t care, this is more important. This person can’t sit there covered in urine or soiled until you’ve finished doing that. These situations are hard to deal with, I don’t know how to handle them”.

(I5)

Responsibility and autonomy are characteristics of a competent professional. Being able to make decisions on the best care for patients constitutes a core nursing competence, as well as a nurse responsibility. A broad majority of nurses who were interviewed showed confidence in care delivery; however, they did not feel this way when they joined the world of work, and needed the help of expert nurses in order to make decisions about problem solving and getting organized at work:

“At first it was a shock. We’re used to spending three years standing behind someone, and whenever a problem crops up we can just ask them, because the responsibility doesn’t fall to us. Then we go to our first day of work and think, ‘Where’s my tutor? I don’t know what to do!’ But then again, expert nurses usually help us”.

(I7)

## 4. Discussion

Theoretical knowledge is the foundation on which practical knowledge is built. Nevertheless, most of the participants expressed having finished their studies with gaps in their knowledge regarding wound care, an essential element of their professional practice [28]. Suboptimal wound management and knowledge have also been identified in different contexts, like the UK [29], Finland [30], or the United States [31]. Furthermore, Welsh [32] has recently concluded that there is a need to improve nursing knowledge and skills on this topic, and highlights the importance of more structured wound care education programs. We agree with Maylor [33], who suggests that tissue viability should be established at a curricular level, as nurses are expected to be competent in it.

NQN may well find themselves working in intensive care units (ICUs), yet university curricula do not offer any courses that cover the care and management of an acutely ill patient, even though student nurses may be assigned to do work placements in hospital ICUs. McCalla-Graham & De Gagne [34] also interviewed NQN and found that the knowledge and skills they received during their education were not sufficient to prepare them for working in acute care settings. Additionally, inexperience, a lack of knowledge, and a lack of confidence were found in reflecting reports of nursing students in ICUs [35]. Williams et al. [36] highlight the fact that nursing education providers need to ensure that students are effectively and safely prepared to respond to critical care challenges competently. On the other hand, Charette et al. [37] expose a crucial fact influencing the deployment of competencies of NQN working in ICUs, which is the “orientation programme” prior to being hired. It consists of theoretical classes and 19 days of preceptorship orientation on a unit. Unfortunately, there is an absence of these programs in our context.

It is well known that concerns about mental health are increasing worldwide [38]. A review of the literature carried out by Barry and Ward [39] concluded that nursing curricula need higher quality theoretical and practical learning opportunities. Liu et al. [40] found that undergraduate nurses in the United States and China were inadequately prepared for mental health management. Nurses and students in this study reported frustration and a lack of knowledge regarding mental disorders and their management.

According to the experience of all the participants, the educational content received regarding pharmacology was weak. Similar findings have been identified previously by Ndosi and Newell [41], and even nursing teachers had similar opinions of low satisfaction with the amount of knowledge taught in preregistration programs [42]. Other authors also found unsatisfactory medication knowledge among last-year students and registered nurses [43]. On the other hand, Romero-Collado et al. [44] analyzed the course content relating to pharmacology in Spanish degree programs and argued that they were sufficient; the difference lies in the fact that it was sufficient inasmuch as it allowed nurses to prescribe medications that did not require a doctor’s prescription.

Another dimension explored in the interviews is the lack of importance given to cultural competence. Some of the participants made suggestions about how the course content could be improved: “nursing care of other cultures should be taught”—the same recommendation given by Gil-Estevan and Solano-Ruiz [45]. This willingness to learn more about how to care for people with different cultural background has been explained in a recent Swedish study [46]. These authors have also highlighted the insufficient availability of that learning. Fortunately, many studies are addressing the teaching of cultural competences, as shown by two systematic reviews [47,48].

Handling end-of-life or critical care communication requires skills that our participants recognized that they lacked. This translates into deficient communication with patient and families, something that has also been reported by other authors [49,50].

Leadership plays a central role in nursing practice. The EFN [9] identify nursing leadership as an essential component and competence-guiding nursing activity. According to this study, NQN have not had the opportunity to develop leadership skills. Morrow [51] highlights the need to design teaching strategies that integrate leadership competences into nursing curricula. Educational innovations have subsequently been designed for this purpose [52].

Barriers in inter-professional communication lead to poor patient outcomes [53]. Our participants showed a lack of the knowledge and skills needed to successfully communicate with other health professionals. This issue has been widely documented, and Foronda et al. [53] show in their integrative review that there are still inter-professional challenges in terms of communication.

However, none of the participants seemed to recognize their own responsibility in their learning process, as can be inferred throughout the analysis of the interviews. Responsibility for their own learning outcomes was apparently and entirely delegated to teachers or clinical preceptors.

Finally, the findings of this study with regard to the student nurses’ transition to employment highlight a lack of confidence when taking on responsibility and autonomy, which has a knock-on effect on the decision-making competence established by the EFN [9]. The literature corroborates new graduates’ perception of having low abilities [25] and an insufficient level of competence [14]. In turn, authors like Martinez-Linares et al. [54] suggest the implementation of some proposals in order to improve nursing competency. On the other hand, Benner [55] states that the acquisition and development of competences occurs along a continuum made up of five levels, beginning with novice, passing through advanced beginner, competent, and proficient, before finally reaching the fifth stage, expert. Lima et al. [14] further support Benner’s proposal. Therefore, if we assume these stages of competency, there should not be such a concern about how competent graduate nurses are upon completion of their studies. However, our participants showed concern about the “know” and the “know-how” in the above-mentioned aspects and did not show full satisfaction. Their expectations were higher, and they would have liked to acquire a more expert level of competence after finishing their studies.

### Limitations

The interpretation of these findings needs to be done with caution as the data were derived from a limited number of participants. Therefore, to proceed with changes in the curricula, further research and follow-up studies would be necessary, including professors and clinical preceptors. Another limitation of the present study is that participants’ views on their responsibility for their own learning have not been explicitly explored and it could generate biased results.

## 5. Conclusions

This investigation has offered recipients of a bachelor’s degree in nursing from a Spanish university the opportunity to speak their minds. NQN and fourth-year students have analogous experiences about the training they received and about the nursing competencies in which they find they are deficient. In addition, we consider that they have a professional imperative to make this known and try to fill the knowledge gaps.

Deficiencies in academic competences relating to cultural knowledge and certain aspects of nursing care have been highlighted. Likewise, shortcomings of a more practical nature were found, such as decision-making, communication, leadership, and nursing care (Competences 1, 3, 4, 5, and 6 of the EFN). To translate the results of this research into practice would mean making changes in the curriculum, although further research is necessary involving professors and clinical preceptors to develop a global understanding of the issues raised in this investigation and to reach a satisfactory solution.

## Figures and Tables

**Table 1:** Relevant scientific literature used as a framework for the interview guide.

Bibliography in Reverse Order of Publication Year	Main Threads Used
Edward et al. [23]	Importance of preceptors for facilitating work readiness and clinical exposure.
Meyer et al. [24]	Educational/Professional Satisfaction Scale (EPSS).
ReBueno et al. [16]	Skills Enhancement Program Questionnaire. Clinical Competence Questionnaire.
Beogo et al. [17]	Clinical Nursing Competence Questionnaire (CNCQ-22).
Wangensteen et al. [18]	Norwegian Nurse Competence Scale (NNCS).
European Federation of Nurses Association (EFN) [9]	Breakdown of competency areas according to the EFN Competency Framework.
Takase et al. [25]	The Holistic Nursing Competence Scale. The Work Environment Scale.
CIN Order/2134/2008 [7]	Section 3. Objectives. Competences that students must acquire.

Source: Prepared by the authors.

**Table 2:** Main questions in the interview guide.

Pre-Established Categories	Main Questions
Clinical training	Which practice was your favorite? Can you explain why? What nursing techniques did you practice? Could you practice them all? Were you involved in any difficult emotional situation that a patient/family member was going through? Can you explain how you felt and how you helped them?
Theoretical/Academic training	Are you satisfied with what you learned at a theoretical level? Can you explain your experience? Which subjects do you think are most important? Can you explain why? Would you make any change to the academic curriculum?
Working situation	How do you evaluate your transition from study to work? What kind of difficulties have you experienced working as a nurse? Which circumstances cause you stress in your day-to-day work?

Source: Prepared by the authors.

**Table 3:** Main questions in the interview guide for focus groups (FGs).

Pre-Established Categories	Main Questions
Clinical training	Were you involved in any difficult emotional situation that a patient/family member was going through? Can you explain how you felt and how you helped them? How comfortable would you feel leading a work team?
Theoretical/Academic training	Are you satisfied with what you learned at a theoretical level? Can you explain your experience? Would you make any change to the academic curriculum?

Source: Prepared by the authors.

**Table 4:** Thematic analysis [26] and establishment of trustworthiness [27].

Phases	Description of the Process	Means of Establishing Trustworthiness
1. Familiarising yourself with your data	Transcribing, reading and re-reading the data and jotting down initial ideas	Prolong engagement with data Triangulate different data collection modes Document theoretical and reflective thoughts Document thoughts about potential codes/themes Store raw data in well-organized archives Keep records of all data field notes, transcripts, and reflexive journals
2. Generating initial codes	Systematic coding (line by line) of interesting features of the data. Collating data relevant to code	Peer debriefing Researcher triangulation Reflexive journaling Use of a coding framework Audit trail of code generation Documentation of all team meeting and peer debriefings
3. Searching for themes	Collating codes into potential themes, gathering all data relevant to each potential theme	Researcher triangulation Diagramming to make sense of theme connections Keeping detailed notes about development and hierarchies of concepts and themes
4. Reviewing themes	Checking whether the themes fit the coded extracts and the entire data set, generating a concept map of the analysis	Researcher triangulation Themes and subthemes vetted by team members Test for referential adequacy by returning to raw data
5. Defining and naming themes	Ongoing analysis to refine the specifics of each theme and the overall story the analysis tells, generating clear definitions and names for each theme	Researcher triangulation Peer debriefing Team consensus on themes Documentation of team meetings regarding themes Documentation of theme naming
6. Drafting the report	Selection of vivid, compelling extract examples and a final analysis of selected extracts, once again relating the analysis to the research question and literature, producing a scholarly report of the analysis	Member checking Peer debriefing Describing process of coding and analysis in sufficient detail Ample descriptions of context Description of the audit trail Report on reasons for theoretical, methodological, and analytical choices throughout the entire study

Source: Prepared by the authors.

**Table 5:** Results: Deficient competences according to the classification by the European Federation of Nurses (EFN, 2015).

Competences According to Directive 2013/55/EU	Competency Areas (CA) EFN Competency Framework (2015)	Deficient Competences According to Breakdown of CA	Related Codes
Competence H	1. Culture, ethics and values	To promote and respect human rights and diversity in the light of the physical, psychological, spiritual and social needs of autonomous individuals, taking into account their opinions, beliefs, values and culture (…)	- Ignorance of other cultures and values. - Devaluation of cultural competence.
Competence C	2. Health promotion and prevention, guidance and teaching	Non deficiency found	Non deficiency found
Competence A Competence F	3. Decision-making	To carry out actions, having identified and analyzed problems, that facilitate seeking the most beneficial solution for the patient (…). To apply critical thinking skills and systems approach to problem solving and nursing decision-making in the professional and care delivery context.	- Needing help from experienced nurses. - Feeling of being lost. - Doubts regarding care.
Competence B Competence G	4. Communication and teamwork	To be able to comprehensively communicate, interact and work effectively with colleagues and inter-professional staff, and therapeutically with individuals, families and groups. To independently coordinate care for patient groups and to work in an interdisciplinary way towards the common goal of ensuring quality of care and patient safety.	- Difficulty of inter-professional communication. - Lack of effective communication with patients/families in difficult situations. - Limitations when confronting other professionals. - Inter-professional conflicts.
Competence A Competence G	5. Research, development and leadership	To adapt leadership styles and approaches to different situations concerning nursing, clinical practice and healthcare.	- Unclear understanding of leadership. - Inability to lead a work team.
Competence A Competence E Competence D	6. Nursing Care: Assessment and diagnosis Care planning Nursing intervention Evaluation and quality assessment	To show sufficient knowledge and skills to provide professional and safe care adequate to the health and nursing care needs of the individuals, families and groups to whom the nurse is responsible for providing care (…)	- Ignorance about mental illnesses. - Insufficient knowledge about critical care. - Lack of information about wound care. - Lack of pharmacological knowledge.

Abbreviations: CA, Competency Areas; EFN, European Federation of Nurses. Source: Prepared by the authors.
